# Nævus

**Published:** 1887-04

**Authors:** 


**Nævus.**—Dr. W. J. Beatty, (*British Medical Journal*) has cured eight cases of nævus, perfectly and painlessly, by painting the effected spot night and morning with liquor arsenicalis until ulceration took place. A cure is effected in from three to five weeks. There may be danger of poisoning in the treatment.—*American Medical Digest.*

